# *Bacillus subtilis* HelD, an RNA Polymerase Interacting Helicase, Forms Amyloid-Like Fibrils

**DOI:** 10.3389/fmicb.2018.01934

**Published:** 2018-08-21

**Authors:** Gundeep Kaur, Srajan Kapoor, Krishan G. Thakur

**Affiliations:** Structural Biology Laboratory, G. N. Ramachandran Protein Centre, Council of Scientific and Industrial Research-Institute of Microbial Technology, Chandigarh, India

**Keywords:** *Bacillus subtilis*, amyloid fibrils, intracellular amyloids, HelD, SEC-SAXS, oligomerization

## Abstract

HelD, an RNA polymerase binding protein from *Bacillus subtilis*, stimulates transcription and helps in timely adaptation of cells under diverse environmental conditions. At present, no structural information is available for HelD. In the current study, we performed size exclusion chromatography coupled to small angle X-ray scattering (SEC-SAXS) which suggests that HelD is predominantly monomeric and globular in solution. Using combination of size exclusion chromatography and analytical ultracentrifugation, we also show that HelD has a tendency to form higher order oligomers in solution. CD experiments suggest that HelD has both α-helical (∼35%) and β sheet (∼26%) secondary structural elements. Thermal melting experiments suggest that even at 90°C, there is only about 30% loss in secondary structural contents with T_m_ of 44°C. However, with the increase in temperature, there was a gain in the β-sheet content and significant irreversible loss of α-helical content. Using a combination of X-ray fiber diffraction analysis, and dye based assays including Thioflavin-T based fluorescence and Congo red binding assays, we discovered that HelD forms amyloid-like fibrils at physiologically relevant conditions *in vitro*. Using confocal imaging, we further show that HelD forms amyloid inclusions in *Escherichia coli*. Bioinformatics-based sequence analysis performed using three independent web-based servers suggests that HelD has more than 20 hot-spots spread across the sequence that may aid the formation of amyloid-like fibrils. This discovery adds one more member to the growing list of amyloid or amyloid-like fibril forming cytosolic proteins in bacteria. Future studies aimed at resolving the function of amyloid-like fibrils or amyloid inclusions may help better understand their role, if any, in the bacterial physiology.

## Introduction

Transcription is an essential and highly regulated complex multi-step process. In bacteria, this process is performed by a single multi-subunit RNA polymerase (RNAP) (Werner, 2008) which interacts with several components of the transcription machinery at various stages of transcription (Murakami, 2015; Browning and Busby, 2016). HelD (also known as YvgS), is one such RNAP binding protein in *Bacillus subtilis* which belongs to the superfamily I of DNA and RNA helicases (Delumeau et al., 2011; Wiedermannova et al., 2014). Helicases are known to interact with the replication and DNA repair machinery (Tuteja and Tuteja, 2004), but recent reports unravel their interactions with the transcription machinery as well, especially with RNAP (Gwynn et al., 2013; Epshtein et al., 2014; Wiedermannova et al., 2014; Sanders et al., 2017). These studies suggest that helicases are diverse enzymes which play important roles in maintaining the genome stability and help in resolving the conflicts between replication and transcription (Yang, 2010). *Escherichia coli* UvrD (Epshtein et al., 2014) and *Geobacillus stearothermophilus* PcrA (Gwynn et al., 2013; Sanders et al., 2017), homologs of *B. subtilis* HelD has been reported to interact with RNAP, thereby, coupling replication and DNA repair with transcription. Both UvrD and PcrA bind and facilitate the back-tracking of RNAP, thus, recruiting nucleotide excision repair machinery, hence, playing an important role in transcription-coupled DNA repair (Gwynn et al., 2013; Epshtein et al., 2014; Sanders et al., 2017).

HelD has a putative three-domain architecture consisting of an N-terminal domain (1–204), a middle domain having ATPase activity (205–606, ATP binding box 220–258) and a C-terminal domain (607–774) (Wiedermannova et al., 2014). The functions of the NTD and CTD of HelD are not yet known. Wiedermannova et al. (2014), reported that HelD binds RNAP near the secondary channel and α-subunit, and increases the rate of transcript formation in an ATP-dependent manner. Though HelD is non-essential, it is required by the cells to adapt to the changing environments (Wiedermannova et al., 2014). HelD is mainly expressed during the stationary phase of growth (Nicolas et al., 2012) and its deletion increases the lag phase and affects the growth of the cells (Wiedermannova et al., 2014). HelD acts synergistically with δ-subunit of RNAP to stimulate transcription and release RNAP from DNA, thus, playing a crucial role in the elongation phase of transcription (Wiedermannova et al., 2014).

HelD has not been extensively studied and at present, no structural information is available. Here, we show that HelD exists predominantly as a monomer in solution and has a tendency to self-associate to form higher order oligomers in solution. While we were characterizing HelD, we serendipitously observed that it possesses an interesting amyloidogenic property. We demonstrate that HelD forms amyloid-like fibrils at physiologically relevant conditions *in vitro* and forms amyloid inclusion *in vivo*. Based on our results, we report that HelD is the first helicase as well as the first soluble intracellular transcription elongation factor and RNAP binding protein in *B. subtilis* to exhibit the amyloidogenic property.

## Materials and Methods

### Cloning, Overexpression, and Purification of HelD

The gene encoding HelD was PCR amplified using gDNA of *B. subtilis* and digested with NdeI and XhoI restriction enzymes. The digested product was then cloned into an expression vector pET28a (Novagen) encoding a Tobacco Etch Virus (TEV) protease cleavable His_6_ tag at the N-terminus, followed by transformation in DH5α competent cells. Initial screening was performed using colony PCR and the positive clones were further confirmed by DNA sequencing. The positive clones were transformed into *E. coli* BL21 (DE3) cells and incubated at 37°C for 12 h. A single colony was used for inoculation in 20 mL LB medium with 50 μg/mL kanamycin at 37°C at a constant speed of 200 rpm. The secondary culture containing kanamycin was set up using primary culture which was further incubated at 37°C with a constant speed of 200 rpm. When the OD_600_ of the cells reached ∼0.5–0.6, protein expression was induced by adding 0.3 mM isopropyl β-D-1- thiogalactopyranoside (IPTG) to the cultures and incubating further at 16°C for 16 h with constant shaking at 200 rpm. The cultures were harvested and cells were resuspended in Buffer A (20 mM HEPES pH 7.5, 150 mM NaCl) along with a cocktail of EDTA-free protease inhibitor tablet (Roche) and then subjected to sonication (10 s “on,” 10 s “off,” 30% amplitude for 30 min). The cell lysate was centrifuged at 18,400 × g for 40 min at 4°C. Thereafter, the supernatant was mixed with 2 mL Co-NTA agarose beads (Gold Biotechnology, St. Louis, MO, United States), pre-equilibrated with Buffer A and kept on a rotator mixer for 30 min at 4°C. The His_6_-tagged HelD was eluted in the Buffer A containing 200 mM imidazole pH 8.0. The fractions were loaded on the 15% SDS-PAGE to check the purity of the samples. The fractions containing purified HelD were pooled and concentrated using 30 kDa cutoff Amicon ultracentrifugation device (Millipore). For the purification of the tagless HelD, the concentrated purified protein was dialyzed in the Buffer A (to remove the imidazole) and subjected to TEV protease cleavage. Purified HelD with His_6_-tag was incubated with TEV protease at 4°C for 8 h. The His_6_-tagged HelD was separated from the tagless HelD by passing the His_6_-HelD-TEV protease mixture through the Co-NTA affinity chromatographic column. The tagless HelD was further purified using size exclusion chromatography (SEC).

### Size Exclusion Chromatography

The oligomeric state of the HelD was determined by SEC using a Superdex 200 Increase 10/300 GL column (GE Healthcare). The column was pre-equilibrated with Buffer A and analytical runs were performed by injecting 500 μL of the purified protein at a flow rate of 0.5 mL/min at 4°C. The fractions corresponding to each peak were pooled and concentrated and later used for structural and biophysical studies.

### Size Exclusion Chromatography Coupled to Small-Angle X-ray Scattering (SEC-SAXS)

SEC-SAXS data for the HelD sample were collected at the Diamond Light Source, Harwell, United Kingdom at B21 beamline. In line SEC-SAXS were performed using an Agilent HPLC equipped with the Shodex column. Data were collected on a Pilatus 2 M detector and HelD (100 μM) was individually loaded onto the column previously equilibrated in Buffer A at a flow rate of 0.05 mL/min. The primary reduction of the SAXS data and data processing were performed using ScÅtter v3.0^1^ to obtain the radius of gyration (R_g_), the maximum particle dimension (D_max_), the excluded particle volume (Vp) and the pair distribution function [P(r)]. The molecular mass of the scattering particles was calculated using a method described by Rambo (Rambo and Tainer, 2013). Low-resolution 3-D *ab initio* models were generated using DAMMIF software (Franke and Svergun, 2009) using slow mode. DAMAVER (Volkov and Svergun, 2003) was used to generate the *ab initio* models. The SAXS envelope was rendered using PyMoL (Schrödinger, LLC, New York, NY, United States). SUPCOMB (Kozin and Svergun, 2001) was used to superpose the homology model into the SAXS based molecular envelope. FoXS server (Schneidman-Duhovny et al., 2010) was used to compute the scattering profile from the *ab initio* generated model and fit with the experimentally observed scattering profile of HelD.

### Homology Modeling

The amino acid sequence of HelD from *B. subtilis* was retrieved from UniProt database and the closest structural homologs were identified using BLAST search against the Protein Data Bank database. The ATPase domain of HelD (residue range 539–641) shares 29% sequence identity with the *E. coli* UvrD. The C-terminal domain (residue range 606–774) shares 39% sequence identity with *Lactobacillus planetarium* (PDB ID 3DMN). No sequence similarity was observed for the N-terminal domain. The homology model was built only for the ATPase and the C-terminal domain using Prime 3.1 module (Jacobson et al., 2004) in Schrödinger software suite (Schrödinger, LLC, New York, NY, United States). SSpro program in Prime was used to predict the secondary structure of HelD. Initially, four models were generated and the model with the least energy was selected for the loop refinement. For refinement of loops comprising of <5 and >5 amino acids, the number of output structures was set to 10 and 5, respectively. After loop refinement, the OPLS3 force field (Harder et al., 2016) was used to minimize the model which was further validated by PROCHECK (Laskowski et al., 1993) for the evaluation of Ramachandran plot (Ramachandran et al., 1963).

### Sedimentation Velocity Analytical Ultracentrifugation (SV-AUC)

SV-AUC experiments were carried out using HelD (5 μM) in the Buffer A at 4°C. Beckman-Coulter XL-A equipped with a TiAn50 eight-hole rotor with two-channel epon centerpiece (12 mm) and quartz window were used to carry out SV experiments. Absorbance scans were recorded at 280 nm at every 4 min interval at 40,000 rpm at 4°C. AUC data was analyzed using the program SEDFIT (Schuck, 2000) by selecting continuous distribution c(s) model. SEDNTERP (Hayes et al., 1995) was used to calculate the solvent density (ρ) and viscosity (η) of the chemical composition of different components present in the Buffer A.

### Circular Dichroism (CD) Spectroscopy

HelD was dialyzed in 10 mM sodium phosphate buffer (pH 7.5) and spectra were collected using a 2 mm path-length cuvette in the wavelength range 190–250 nm. The CD experiments were recorded at 5 μM protein concentration using a JASCO J-810 spectrometer and analyzed using OriginPro 2016 software. Thermal melt experiments were performed by increasing the temperature from 20 to 90°C with a ramp rate of 1°C/min. The spectra were collected at every 5°C with a scanning speed of 100 nm/min. The melting curves were plotted and T_m_ of the sample was calculated using OriginPro 2016 software.

### Monitoring Kinetics and Formation of HelD Amyloid-Like Fibrils *in vitro*

Thioflavin T (ThT) fluorescence assay was performed by incubating HelD (100 μM) with ThT (1 mM) at 37°C for 5 min. The ThT fluorescence intensity was measured using excitation wavelength of 440 nm and an emission wavelength of 485 nm. The fluorescence measurements were recorded using Synergy H1 Hybrid Multi-mode Micro-plate reader. The kinetics of amyloid-like fibril formation by HelD (100 μM) was monitored at a constant temperature of 37°C and readings were acquired every 2 min over a period of 8 h. The top of each well was sealed using adhesive tape to minimize the rate of evaporation in all the experiments. Three independent experiments were performed in triplicates and the bars represent the standard deviation.

### Congo Red (CR) Binding Assay

The method described by Klunk et al. (1989) was followed for CR binding assay. A solution of 7 mg/mL of CR was prepared in Buffer A and passed through 0.22 micron filter prior to use. The spectrum of CR (5 μL in 1 mL Buffer A) was recorded between 400 and 700 nm at room temperature (control). Varying concentrations of HelD (0.6, 0.8, or 1 μM) were incubated with CR for 30 min at room temperature and the spectrum were recorded between 400 and 700 nm using Cecil 7500 Double Beam UV/Visible spectrophotometer.

### X-ray Diffraction Analysis

The solution containing the preformed fibrils, prepared as described above from 100 μM HelD solution, were centrifuged at 5,000 × *g* for 2 min. The supernatant was aspirated and the pellet was resuspended in the remaining solution and vortexed. This sample was transferred to the 0.6 mm borosilicate glass capillary (Hampton Research) and sealed from the narrow end allowing evaporation from the other end. The sample was dried by incubating at 37°C overnight. The X-ray fiber diffraction data were collected at the home source (Rigaku micromax 007 equipped with Mar345 detector) at the wavelength of 1.5418 Å. The air dried sample in the capillary was exposed to the beam at room temperature for 20 min. The diffraction pattern was analyzed using HKL2000 (Otwinowski and Minor, 1997).

### Optical and Confocal Microscopic Visualization of Amyloid-Like Fibrils

Ten microliter of 100 μM HelD was dispensed on the glass slide, air-dried and stained with ThT (1 mM). The excess stain was blotted away and the slide was further air-dried. The slides were visualized using Olympus microscope model BX51, using a 100× oil-immersion objective under bright field and cross-polarized light. The images were captured using cellSens software. The slides were also visualized using confocal fluorescence microscope (Nikon A1R), using a 100× oil-immersion objective and 1 Airy unit aperture. The ThT stained samples were excited using an excitation wavelength of 440 nm and an emission wavelength of 480 nm.

### Thioflavin S (ThS) Staining of the Live Bacterial Cells

ThS staining of the live bacterial cells was performed as per the protocol mentioned by Kaur et al. (2018). Briefly, cells harboring HelD and empty expression vector were grown in LB media at 37°C until OD_600_ reached 0.5. The protein expression was then induced using 0.3 mM IPTG and incubated for 5 h. The 0.5 mL of culture was then centrifuged at 6,000 × g at 25°C for 2 min. followed by 3 times washing with 1× PBS. The cells were fixed by adding 4% paraformaldehyde and incubating at 37°C for 30 min. Cells were again washed three times with 1× PBS. Fixed cells were then incubated with 250 μL of 0.05% (w/v) ThS (Catalog number T1892, Sigma) prepared in 12.5% ethanol at 37°C for 30 min in the dark (Aguilera et al., 2016). Stained cells were again washed three times with 1× PBS. Ten microliter of sample was then placed on the top of the glass slide, air-dried and covered with cover slip. The images were acquired using confocal fluorescence microscope (Nikon A1R), using a 100× oil-immersion objective and 1.2 Airy unit aperture. The ThS stained samples were excited using an excitation wavelength of 405 nm and an emission wavelength of 482 nm.

## Results

### HelD Has a Propensity to Form Higher Order Oligomers *in vitro*

**FIGURE 1:**
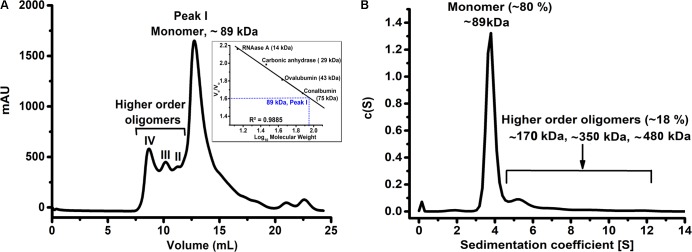
HelD has a tendency to form higher order oligomers in solution. **(A)** The SEC profile of HelD showing the predominant monomer (peak I) population as well as minor peaks II-IV corresponding to the higher order oligomers in solution. **(B)** The SV-AUC analysis of HelD shows that HelD exists predominantly as a monomer (∼80% population) and has a tendency to oligomerize (∼20% population) in solution.

We successfully purified HelD from the soluble fraction suitable for structural and biophysical studies. The purity and molecular weight of HelD were assessed by 15% SDS-PAGE (**Supplementary Figure 1**). As the oligomeric state of HelD was not known so we performed analytical SEC experiments. The analytical SEC profile of HelD revealed that HelD has a tendency to exist in multiple distinct oligomeric states in solution. The theoretical molecular weight of HelD is 90 kDa and we could observe a predominant peak corresponding to monomer [Peak I, observed molecular weight (MW_obs_) ∼89 kDa] and minor peaks corresponding to dimer, trimer and higher order oligomeric species of HelD in solution (**Figure 1A**). Further, SV-AUC results correlate well with the SEC studies. The SV-AUC profile suggests that HelD exists predominantly as a monomer (MW_obs_ = 89 kDa, ∼80%) and has the tendency to self-associate to form higher order oligomers (∼20% population; **Figure 1B**) in solution. The Stokes radius obtained from the AUC experiments is 3.55 nm and the value of the best-fit frictional ratio is 1.2, which suggests globular state of HelD.

### SEC-SAXS Based Low-Resolution Solution Structure of HelD

**FIGURE 2:**
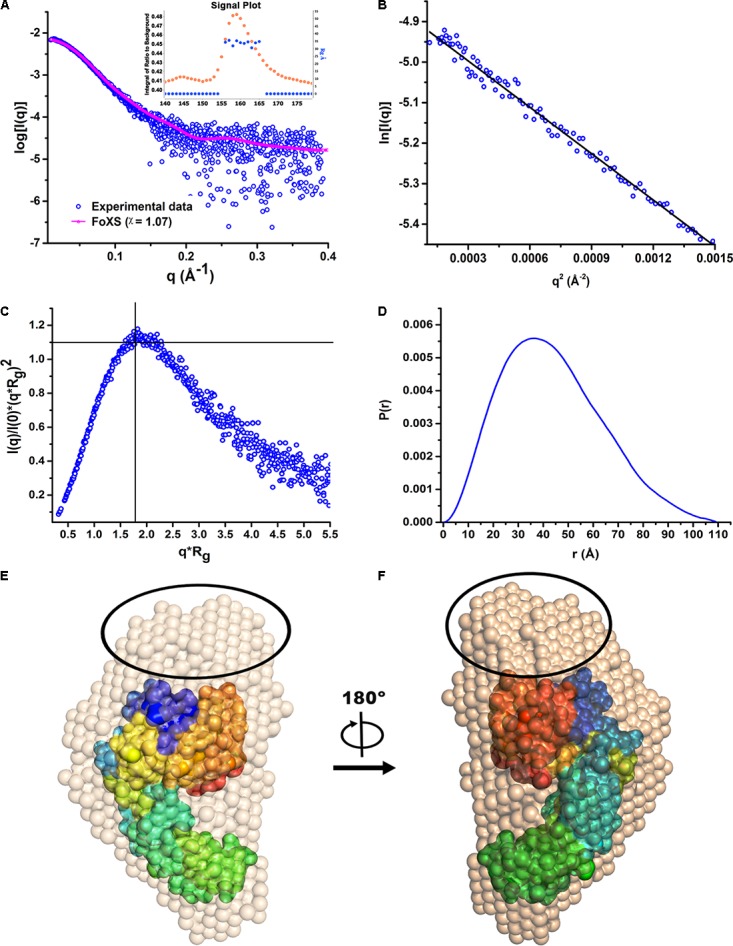
The low-resolution solution structure of HelD. **(A)** The SAXS intensity profile of the monomeric HelD. The R_g_ values are consistent across the predominant peak observed in the SEC profile (Inset). Superposition of the experimental scattering profile (blue) with the theoretically computed scattering profile (magenta) of the *ab initio* model using FoXS web server (χ = 1.04) suggests good fit. **(B)** The low-q region (0.02–0.19) used for the Guinier analysis. **(C)** The Kratky analysis suggests HelD as perfectly globular and well-folded in solution. **(D)** Normalized pair-wise distribution function [P(r)] suggest D_max_ of 97 Å. **(E,F)** The *in silico* generated homology model of HelD lacking the NTD was superposed into the SAXS-derived dummy atom model generated by DAMMIF using the program SUPCOMB (NSD = 2.6). The unaccounted region in the SAXS derived envelope, possibly corresponding to NTD, is encircled in black. Two different views of the superposed models rotated counter-clock wise, around the vertical y-axis are shown.

Since there was no structural information available for HelD, so we initiated crystallization trials. Our crystallization experiments did not yield reproducible diffraction quality crystals. In order to gain structural insights of HelD in solution, we performed SEC-SAXS experiments to obtain a low-resolution structural model of HelD. The primary data reduction and data processing of the sample (**Figure 2A**) were performed using ScÅtter v3.0^2^. The scattering intensity profile, Guinier plot, Kratky plot and Pairwise distribution function [P(r)], are all plotted in **Figures 2A–D**, respectively. The data was analyzed over the q range 0.02–0.19 Å^−1^ (**Figure 2A**). The analysis of the signal plot suggests that values of the radius of gyration (R_g_) are consistent across the peak (**Figure 2A**, inset). The analysis of the Guinier plot suggests a linear fit with no apparent signs of aggregation (**Figure 2B**). Kratky plot analysis suggests that HelD is globular in solution as the peak is located at the ideal position in the Kratky plot (**Figure 2C**). P(r) analysis suggests a maximum dimension, D_max_ of 97 Å (**Figure 2D**). The R_g_ calculated from the reciprocal space (34.12 ± 0.051 Å) matches well with the R_g_ obtained from the real space (33.56 ± 0.068 Å). The real space R_g_ obtained from SAXS (3.35 nm) is in close agreement with the Stokes radius obtained from the AUC experiments (3.55 nm). The molecular weight (MW) was calculated by dividing the Porod volume (164,764 Å^3^) by 1.7. The calculated MW of 96.9 kDa is in close agreement with the theoretically expected molecular weight, MW_exp_ of 90 kDa. Hence, the predominant peak in the SEC profile corresponds to the monomeric state of HelD. The *ab initio* models generated by DAMMIF, further averaged using DAMAVER, suggest that HelD adopts a C-shaped globular architecture in solution (**Figures 2E,F**).

As no experimental structural information was available, so we also performed *in silico* homology modeling using Prime module of Schrödinger suite. The final generated homology model was superposed to the averaged molecular envelope of dummy atom model using SUPCOMB (Kozin and Svergun, 2001; **Figures 2E,F**). The homology model fits well in the SAXS envelope (NSD = 2.6). FoXS server (Schneidman-Duhovny et al., 2010) was used to align and match the experimentally observed scattering profile with the theoretically obtained scattering profile from the dummy atom model, which suggests a good fit (χ = 1; **Figure 2A**). An unaccounted region in the SAXS derived molecular envelop was observed which may correspond to the N-terminal domain of HelD which is absent in the homology model (**Figures 2E,F**).

### Secondary Structural Analysis and Thermal Melting Studies of HelD

**FIGURE 3:**
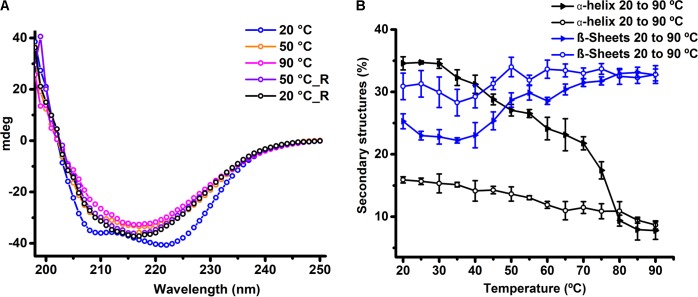
HelD retains β-sheet structures upon thermal denaturation in solution. **(A)** CD spectra of HelD collected as a function of temperature. HelD was heated from 20 to 90°C and then cooled from 90 to 20°C. For clarity, the representative CD spectra acquired at 20°, 50°, and 90°C during both heating and cooling (represented with “_R”) are plotted. **(B)** BeStSel webserver based analysis of secondary structural changes in the HelD upon heating and cooling experiments. The analysis reveals the structural transitions accompanied by loss of α-helical content with increase in the β-sheet contents upon heating. The protein did not attain its native secondary structural contents upon cooling.

To estimate the secondary structural contents in HelD, we performed CD spectroscopy experiments. BeStSel webserver (Micsonai et al., 2015) was used to deconvolute and estimate the secondary structural content from the acquired CD spectra. Our experiments performed at 20°C suggests that HelD is composed of a mix of α-helical (35%) and β-sheet (26%) secondary structural elements (**Figure 3A**). We also assessed the thermal stability of HelD using CD-based thermal denaturation experiments. Thermal denaturation profile suggests that HelD undergoes partial thermal unfolding with a T_m_ of 44°C (**Supplementary Figure 2**). With the increase in the temperature (20–90°C), we could clearly observe the structural transitions accompanied by significant loss of signatures corresponding to α-helix (negative dip at 222 and 208 nm) while there was a gain in the characteristic signatures corresponding to the β-sheet (negative dip at 218 nm) (**Figure 3A**). The deconvolution of the CD spectra with respect to change in temperature using the BeStSel webserver (Micsonai et al., 2015) suggests about 80% decrease in the α-helical content and approximately 20% increase in the β-sheet content (**Figure 3B**). Interestingly, even at 90°C, there is only a loss of about 30% secondary structural contents. However, when the temperature was reduced from 90° to 20°C, HelD did not attain its native structure in solution. We did not observe any visible precipitation of the sample upon heating and cooling, so we were interested in knowing whether HelD sample was still predominantly monomeric in solution. We performed SEC experiments by injecting this heat treated sample in the Superdex 200 Increase 10/300 GL column. We observed major peak eluting at the void volume suggesting presence of higher order oligomeric species or soluble aggregates (**Supplementary Figure 3**). This data suggests that protein partially unfolds upon heating and probably either forms soluble aggregates or self-associate to form higher order oligomeric species with substantial increase in β structure.

### HelD Forms Amyloid-Like Fibrils in Solution

There are several reports in the literature which suggest a strong correlation between the protein oligomerization, the presence of intrinsically disordered segments and amyloid formation (Tompa, 2009; Kumar and Udgaonkar, 2010; Fandrich, 2012). The ability of HelD to exist in multiple oligomeric states in solution, the presence of intrinsically disordered regions (as predicted using CamSol; Sormanni et al., 2015a) and the increase in the β-sheet content on thermal denaturation, prompted us to investigate its amyloidogenic property.

To begin with, we performed a bioinformatics-based sequence analysis to predict the presence of amyloid-forming regions using PASTA 2.0 web server (Walsh et al., 2014), Aggrescan (Conchillo-Sole et al., 2007) and FoldAmyloid (Garbuzynskiy et al., 2010). PASTA predicted 20 amyloid structural aggregation regions in HelD and the best aggregation pairing energy was −7.95. Aggrescan predicted 25 hot spots of aggregation and FoldAmyloid predicted 28 amyloidogenic regions in HelD. The predicted regions are not restricted to a specific region and are spanned over the whole sequence. Most of the predicted amyloidogenic regions are having a significant overlap suggesting consistency in the bioinformatics based predictions. The bioinformatics results and analysis are summarized in the **Supplementary Figure 4**.

**FIGURE 4:**
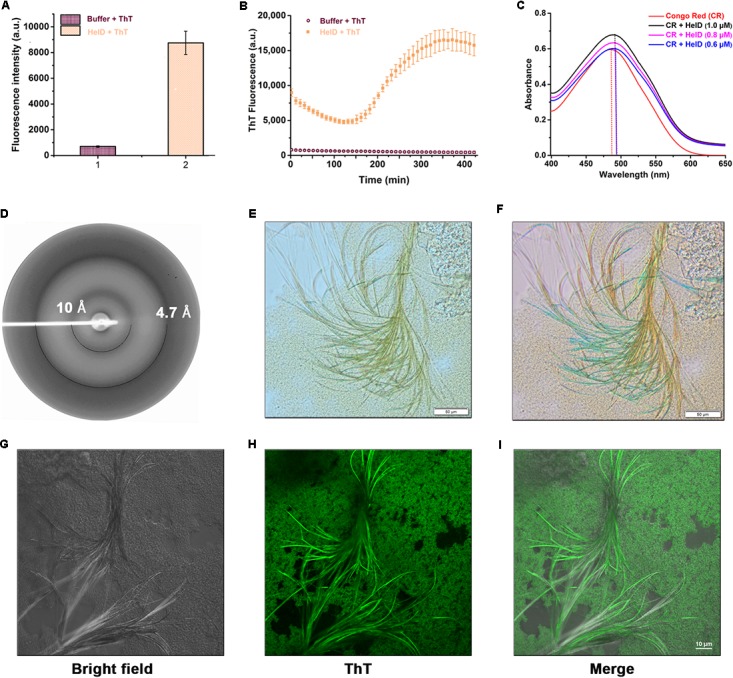
HelD forms amyloid-like fibrils *in vitro.* **(A)** Incubating HelD with ThT at 37°C for 5 min shows enhanced fluorescence intensity. The average fluorescence intensity and standard error bars have been calculated from the three independent experiments. **(B)** The kinetics of HelD (100 μM) amyloid fibril formation monitored by ThT fluorescence emission. **(C)** Congo red absorbance spectra in the absence and presence of varying concentrations of HelD (0.6, 0.8, and 1 μM) suggests a red shift in the absorbance maxima of CR in the presence of HelD. **(D)** X-ray diffraction pattern of HelD amyloid-like fibrils showing reflections at ∼4.7 and ∼10 Å. **(E,F)** Optical microscopy image of HelD amyloid-like fibrils stained positive with ThT; under bright light **(E)** and yellow-green birefringence observed under the cross-polarized light **(F)**. **(G–I)** Confocal microscopy images of HelD amyloid-like fibrils stained positive with ThT. Left, middle and right panels represent the bright field, ThT-stained and merged images, respectively.

We then performed a combination of amyloid specific assays [Thioflavin T (ThT) fluorescence- and microscopy-based assays, Congo Red (CR) binding assay and X-ray fiber diffraction analysis] to investigate whether HelD forms amyloid-like fibrils *in vitro* as suggested by the preliminary bioinformatics based predictions. ThT is a fluorescent dye which binds specifically to the amyloid fibrils *in vitro* (Saeed and Fine, 1967) and gives a strong fluorescence signal at about 482 nm when excited at 440 nm. We incubated HelD with ThT at 37°C for 5 min and observed a significant increase in the fluorescence intensity as compared with the control sample, thus, suggesting the presence of amyloid-like fibrils in HelD (**Figure 4A**). Further, we monitored the kinetics of the formation of amyloid-like fibrils at 37°C for a period of 8 h. We observed a characteristic sigmoidal curve consisting of a lag phase, the elongation phase as well as stationary phase (**Figure 4B**). We also performed Congo red (CR) binding assay (**Figure 4C**) in the absence and presence of varying concentrations of HelD (0.6, 0.8, and 1 μM). HelD fibrils displayed a red-shift in the CR binding assay, a characteristic feature of amyloid fibrils (Klunk et al., 1989). The X-ray diffraction pattern of HelD amyloid fibrils exhibited two different reflections, at ∼4.7 and ∼10 Å (**Figure 4D**). Both the reflections correspond to the cross-β pattern, characteristic of amyloid diffraction hence indicating amyloid-like fibril nature of HelD fibrils (Morris and Serpell, 2012). We also performed optical microscopy to visualize and analyze the morphology of the HelD amyloid-like fibrils, stained positive with ThT (**Figures 4E,F**). We could observe yellow colored several micron long fibrils when viewed in the bright field microscope (**Figure 4E**). The fibrils showed yellowish-green birefringence, a characteristic feature of amyloid fibrils, when viewed under the cross-polarized light (**Figure 4F**). In order to re-confirm and visualize the presence of amyloid-like fibrils in solution, we performed confocal microscopy imaging experiments where we could observe the green fluorescent amyloid-like fibrils of HelD stained with ThT (**Figures 4G–I**). All these *in vitro* results suggest that HelD has a tendency to form amyloid-like fibrils in solution at physiologically relevant conditions.

### HelD Forms Amyloid Inclusions *in vivo*

**FIGURE 5:**
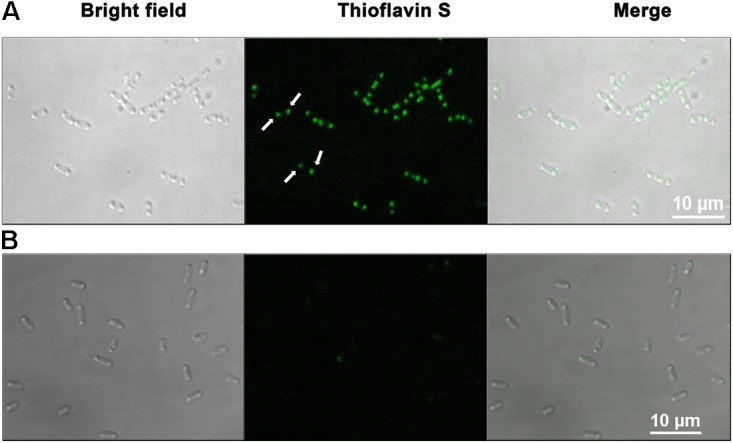
HelD forms amyloid inclusions in *E. coli*. ThS staining in intact bacterial cells **(A)** overexpressing HelD reveal the presence of green colored amyloid inclusions (indicated by white arrows) at the poles in the cells, and **(B)** background fluorescence in the induced control cells carrying empty expression vector, when viewed using confocal microscope at 100× magnification.

After establishing that HelD forms amyloid-like fibrils *in vitro* we were interested to investigate whether it has a tendency to form amyloid-inclusions *in vivo* as well. Thioflavin S (ThS), a cell-permeable dye, is commonly used to stain the intracellular amyloid-like aggregates in bacteria (Espargaro et al., 2012; Pouplana et al., 2014; Macedo et al., 2015; Aguilera et al., 2016; Batlle et al., 2017). We also followed a similar strategy, where we stained *E. coli* cells, either overexpressing HelD or harboring the empty expression vector (control), using ThS and later visualized using confocal microscopy. Both the control and the HelD overexpressing cells were processed using the same protocol. Strong green fluorescence was observed near the poles of the *E. coli* cells over-expressing HelD, thus, suggesting the presence of amyloid inclusions in the bacteria (**Figure 5**). The *E. coli* cells carrying empty expression vector exhibited only residual fluorescence. Several proteins have been reported to form amyloid inclusions in bacteria (de Groot et al., 2009; Rinas et al., 2017; Kaur et al., 2018). Both *in vitro* and *in vivo* data suggest that HelD has a tendency to form amyloid-like fibrils.

## Discussion

In the present study, while characterizing a helicase and a bacterial RNAP binding protein, HelD, we serendipitously observed its amyloidogenic property. SEC-SAXS data analysis suggests that HelD is a soluble globular protein which exists predominantly as a monomer in solution but has a tendency to oligomerize and form higher order oligomers in solution. Interestingly, we also observed that with the increase in temperature, HelD undergoes significant irreversible structural transitions, accompanied by loss of α-helical content and gain of β-sheet content. Further analysis suggests that upon heating HelD either forms higher order oligomers or soluble aggregates which may partly explain the inability to regain native like conformation, though there is only about 10% loss in secondary structural content at 90°C. *Mycobacterium tuberculosis* CarD, on the other hand, has been recently discovered to form amyloid-like fibrils which undergo reversible structural changes upon heating (Kaur et al., 2018). The presence of polyproline-II elements in the proteins has been implicated in amyloidogenesis (Adzhubei et al., 2017). Interestingly, the s2D method (Sormanni et al., 2015b) predicts the presence of polyproline-II elements in HelD (**Supplementary Figure 5**). Surprisingly, we discovered that HelD also displays an unusual ability to form amyloid-like fibrils at physiologically relevant temperatures *in* *vitro* and forms amyloid inclusions *in* *vivo* in *E. coli*. Generally, amyloid fibrils are unbranched. However, microscopy data suggests that HelD probably forms branched amyloid-like fibrils. There are several examples in the literature where proteins reportedly form branched amyloid fibrils (Morozova-Roche et al., 2000; Del Mercato et al., 2007; Andersen et al., 2009; Pedersen et al., 2010; Agopian and Guo, 2012; Liu et al., 2016; Sulatskaya et al., 2017). Based on the data presented here, we report that HelD is the first helicase as well as the first soluble intracellular transcription elongation factor and RNAP binding protein in *B. subtilis* to exhibit the amyloidogenic property. In past several proteins have been shown to form amyloid-like fibrils under non-physiological conditions like heating, extreme pH etc. TasA, an extracellular protein, is required for the structural integrity of biofilm in *B. subtilis* (Branda et al., 2001; Romero et al., 2010b). It has been shown to exist in multiple oligomeric states *in vitro* and has an ability to form amyloid fibrils (Diehl et al., 2018). Recently, based on the X-ray diffraction analysis it has been shown that TasA forms functional fibrils which are non-amyloidogenic in nature (Erskine et al., 2018).

Amyloid-forming proteins have been discovered in all domains of life ranging from bacteria to eukaryotes. Usually, amyloids are associated with eukaryotic proteins and are involved in neurodegenerative disorders (Wolfe and Cyr, 2011). However, amyloidogenesis has not been extensively studied in microbes. The research in this area has accelerated in recent times with discovery of several cytosolic bacterial proteins such as *E. coli* RavA (Chan et al., 2016), *E. coli* GroES (Higurashi et al., 2005), *E. coli* RepA (Giraldo, 2007; Fernández et al., 2016), Microcin E492 (Aguilera et al., 2016), *Clostridium botulinum* Rho (Pallares et al., 2015; Yuan and Hochschild, 2017) and *M. tuberculosis* CarD (Kaur et al., 2018) added to the list of family of proteins with amyloidogenic properties. Our study provides the first report of the formation of amyloid-like fibrils by a soluble cytoplasmic transcription factor, HelD, in *B. subtilis* which also functions as a helicase. There are some recent reports that suggest a role of functional bacterial amyloids which play a crucial role in promoting host-pathogen interactions (Cherny et al., 2005; Alteri et al., 2007; Romero et al., 2010a; Quay et al., 2015; Diehl et al., 2018).

HelD is also present in the pathogenic *Bacillus* species including *Bacillus anthracis*, *Bacillus thuringiensis* and *Bacillus cereus.* Sequence-based bioinformatics analysis using MetAmyl (Emily et al., 2013) suggests that HelD homologs in pathogenic species have a higher number of amyloid fragments/hot spots for amyloidogenic regions as compared to *B. subtilis* HelD (**Supplementary Figure 6**). Future studies aimed at determining the structure of HelD in monomeric and amyloid-like fibril states may help resolve the structural transitions that promote amyloidogenesis. It will be interesting to investigate whether HelD forms amyloid inclusions *in vivo* in *B. subtilis* and elucidate the physiological role(s) played by HelD amyloids, if any, in the bacterial pathogenesis, gene regulation, and/or viability.

## Author Contributions

GK and KT proposed the concepts, designed the experiments, and wrote the manuscript. GK and SK carried out the experiments. GK, SK, and KT analyzed and interpreted the results.

## Conflict of Interest Statement

The authors declare that the research was conducted in the absence of any commercial or financial relationships that could be construed as a potential conflict of interest.

## Footnotes

1 http://www.bioisis.net/

2 http://www.bioisis.net/

## References

[B1] Adzhubei A. A. Anashkina A. A. Makarov A. A. (2017). Left-handed polyproline-II helix revisited: proteins causing proteopathies. *J. Biomol. Struct. Dyn.* 35 2701–2713. 10.1080/07391102.2016.1229220 27562438

[B2] Agopian A. Guo Z. (2012). Structural origin of polymorphism of Alzheimer’s amyloid beta-fibrils. *Biochem. J.* 447 43–50. 10.1042/BJ20120034 22823461

[B3] Aguilera P. Marcoleta A. Lobos-Ruiz P. Arranz R. Valpuesta J. M. Monasterio O. (2016). Identification of key amino acid residues modulating intracellular and in vitro microcin E492 amyloid formation. *Front. Microbiol.* 7:35. 10.3389/fmicb.2016.00035 26858708 PMC4729943

[B4] Alteri C. J. Xicohténcatl-Cortes J. Hess S. Caballero-Olín G. Girón J. A. Friedman R. L. (2007). Mycobacterium tuberculosis produces pili during human infection. *Proc. Natl. Acad. Sci. U.S.A.* 104 5145–5150. 10.1073/pnas.0602304104 17360408 PMC1817835

[B5] Andersen C. B. Yagi H. Manno M. Martorana V. Ban T. Christiansen G. (2009). Branching in amyloid fibril growth. *Biophys. J.* 96 1529–1536. 10.1016/j.bpj.2008.11.024 19217869 PMC2717229

[B6] Batlle C. de Groot N. S. Iglesias V. Navarro S. Ventura S. (2017). Characterization of soft amyloid cores in human prion-like proteins. *Sci. Rep.* 7:12134. 10.1038/S41598-017-09714-Z 28935930 PMC5608858

[B7] Branda S. S. Gonzalez-Pastor J. E. Ben-Yehuda S. Losick R. Kolter R. (2001). Fruiting body formation by *Bacillus subtilis*. *Proc. Natl. Acad. Sci. U.S.A.* 98 11621–11626. 10.1073/pnas.191384198 11572999 PMC58779

[B8] Browning D. F. Busby S. J. (2016). Local and global regulation of transcription initiation in bacteria. *Nat. Rev. Microbiol.* 14 638–650. 10.1038/nrmicro.2016.103 27498839

[B9] Chan S. W. Yau J. Ing C. Liu K. Farber P. Won A. (2016). Mechanism of amyloidogenesis of a Bacterial AAA+ chaperone. *Structure* 24 1095–1109. 10.1016/j.str.2016.05.002 27265850

[B10] Cherny I. Rockah L. Levy-Nissenbaum O. Gophna U. Ron E. Z. Gazit E. (2005). The formation of *Escherichia coli* curli amyloid fibrils is mediated by prion-like peptide repeats. *J. Mol. Biol.* 352 245–252. 10.1016/j.jmb.2005.07.028 16083908

[B11] Conchillo-Sole O. de Groot N. S. Aviles F. X. Vendrell J. Daura X. Ventura S. (2007). AGGRESCAN: a server for the prediction and evaluation of “hot spots” of aggregation in polypeptides. *BMC Bioinformatics* 8:65. 10.1186/1471-2105-8-65 17324296 PMC1828741

[B12] de Groot N. S. Sabate R. Ventura S. (2009). Amyloids in bacterial inclusion bodies. *Trends Biochem. Sci.* 34 408–416. 10.1016/j.tibs.2009.03.009 19647433

[B13] Del Mercato L. L. Pompa P. P. Maruccio G. Della Torre A. Sabella S. Tamburro A. M. (2007). Charge transport and intrinsic fluorescence in amyloid-like fibrils. *Proc. Natl. Acad. Sci. U.S.A.* 104 18019–18024. 10.1073/pnas.0702843104 17984067 PMC2084289

[B14] Delumeau O. Lecointe F. Muntel J. Guillot A. Guedon E. Monnet V. (2011). The dynamic protein partnership of RNA polymerase in *Bacillus subtilis*. *Proteomics* 11 2992–3001. 10.1002/pmic.201000790 21710567

[B15] Diehl A. Roske Y. Ball L. Chowdhury A. Hiller M. Moliere N. (2018). Structural changes of TasA in biofilm formation of *Bacillus subtilis*. *Proc. Natl. Acad. Sci. U.S.A.* 115 3237–3242. 10.1073/pnas.1718102115 29531041 PMC5879678

[B16] Emily M. Talvas A. Delamarche C. (2013). MetAmyl: a METa-predictor for AMYLoid proteins. *PLoS One* 8:e79722. 10.1371/journal.pone.0079722 24260292 PMC3834037

[B17] Epshtein V. Kamarthapu V. McGary K. Svetlov V. Ueberheide B. Proshkin S. (2014). UvrD facilitates DNA repair by pulling RNA polymerase backwards. *Nature* 505 372–377. 10.1038/nature12928 24402227 PMC4471481

[B18] Erskine E. Morris R. J. Schor M. Earl C. Gillespie R. M. C. Bromley K. (2018). Formation of functional, non-amyloidogenic fibres by recombinant *Bacillus subtilis* TasA. *Mol. Microbiol.* 10.1111/mmi.13985 [Epub ahead of print]. 29802781 PMC6334530

[B19] Espargaro A. Sabate R. Ventura S. (2012). Thioflavin-S staining coupled to flow cytometry. A screening tool to detect in vivo protein aggregation. *Mol. Biosyst.* 8 2839–2844. 10.1039/c2mb25214g 22868714

[B20] Fandrich M. (2012). Oligomeric intermediates in amyloid formation: structure determination and mechanisms of toxicity. *J. Mol. Biol.* 421 427–440. 10.1016/j.jmb.2012.01.006 22248587

[B21] Fernández C. Núñez-Ramírez R. Jiménez M. Rivas G. Giraldo R. (2016). RepA-WH1, the agent of an amyloid proteinopathy in bacteria, builds oligomeric pores through lipid vesicles. *Sci. Rep.* 6:23144. 10.1038/srep23144 26984374 PMC4794723

[B22] Franke D. Svergun D. I. (2009). DAMMIF, a program for rapid ab-initio shape determination in small-angle scattering. *J. Appl. Crystallogr.* 42 342–346. 10.1107/S0021889809000338 27630371 PMC5023043

[B23] Garbuzynskiy S. O. Lobanov M. Y. Galzitskaya O. V. (2010). FoldAmyloid: a method of prediction of amyloidogenic regions from protein sequence. *Bioinformatics* 26 326–332. 10.1093/bioinformatics/btp691 20019059

[B24] Giraldo R. (2007). Defined DNA sequences promote the assembly of a bacterial protein into distinct amyloid nanostructures. *Proc. Natl. Acad. Sci. U.S.A.* 104 17388–17393. 10.1073/pnas0702006104 17959784 PMC2077266

[B25] Gwynn E. J. Smith A. J. Guy C. P. Savery N. J. McGlynn P. Dillingham M. S. (2013). The conserved C-terminus of the PcrA/UvrD helicase interacts directly with RNA polymerase. *PLoS One* 8:e78141. 10.1371/journal.pone.0078141 24147116 PMC3797733

[B26] Harder E. Damm W. Maple J. Wu C. Reboul M. Xiang J. Y. (2016). OPLS3: a force field providing broad coverage of drug-like small molecules and proteins. *J. Chem. Theory Comput.* 12 281–296. 10.1021/acs.jctc.5b00864 26584231

[B27] Hayes D. Laue T. Philo J. (1995). *Program Sednterp: Sedimentation Interpretation Program*. Durham, NH: University of New Hampshire.

[B28] Higurashi T. Yagi H. Mizobata T. Kawata Y. (2005). Amyloid-like fibril formation of co-chaperonin GroES: nucleation and extension prefer different degrees of molecular compactness. *J. Mol. Biol.* 351 1057–1069. 10.1016/j.jmb.2005.07.006 16054644

[B29] Jacobson M. P. Pincus D. L. Rapp C. S. Day T. J. Honig B. Shaw D. E. (2004). A hierarchical approach to all-atom protein loop prediction. *Proteins* 55 351–367. 10.1002/prot.10613 15048827

[B30] Kaur G. Kaundal S. Kapoor S. Grimes J. M. Huiskonen J. T. Thakur K. G. (2018). Mycobacterium tuberculosis CarD, an essential global transcriptional regulator forms amyloid-like fibrils. *Sci. Rep.* 8:10124. 10.1038/s41598-018-28290-4 29973616 PMC6031611

[B31] Klunk W. E. Pettegrew J. W. Abraham D. J. (1989). Two simple methods for quantifying low-affinity dye-substrate binding. *J. Histochem. Cytochem.* 37 1293–1297. 10.1177/37.8.2666512 2666512

[B32] Kozin M. B. Svergun D. I. (2001). Automated matching of high- and low-resolution structural models. *J. Appl. Crystallogr.* 34 33–41. 10.1107/S0021889800014126

[B33] Kumar S. Udgaonkar J. B. (2010). Mechanisms of amyloid fibril formation by proteins. *Curr. Sci.* 98 639–656.

[B34] Laskowski R. A. MacArthur M. W. Moss D. S. Thornton J. M. (1993). PROCHECK: a program to check the stereochemical quality of protein structures. *J. Appl. Crystallogr.* 26 283–291. 10.1107/S0021889892009944

[B35] Liu J. Costantino I. Venugopalan N. Fischetti R. F. Hyman B. T. Frosch M. P. (2016). Amyloid structure exhibits polymorphism on multiple length scales in human brain tissue. *Sci. Rep.* 6:33079. 10.1038/srep33079 27629394 PMC5024092

[B36] Macedo B. Sant’Anna R. Navarro S. Cordeiro Y. Ventura S. (2015). Mammalian prion protein (PrP) forms conformationally different amyloid intracellular aggregates in bacteria. *Microb Cell Fact.* 14:174. 10.1186/s12934-015-0361-y 26536866 PMC4634817

[B37] Micsonai A. Wien F. Kernya L. Lee Y. H. Goto Y. Refregiers M. (2015). Accurate secondary structure prediction and fold recognition for circular dichroism spectroscopy. *Proc. Natl. Acad. Sci. U.S.A.* 112 E3095–E3103. 10.1073/pnas.1500851112 26038575 PMC4475991

[B38] Morozova-Roche L. A. Zurdo J. Spencer A. Noppe W. Receveur V. Archer D. B. (2000). Amyloid fibril formation and seeding by wild-type human lysozyme and its disease-related mutational variants. *J. Struct. Biol.* 130 339–351. 10.1006/jsbi.2000.4264 10940237

[B39] Morris K. L. Serpell L. C. (2012). X-ray fibre diffraction studies of amyloid fibrils. *Methods Mol. Biol.* 849 121–135. 10.1007/978-1-61779-551-0_9 22528087

[B40] Murakami K. S. (2015). Structural biology of bacterial RNA polymerase. *Biomolecules* 5 848–864. 10.3390/biom5020848 25970587 PMC4496699

[B41] Nicolas P. Mader U. Dervyn E. Rochat T. Leduc A. Pigeonneau N. (2012). Condition-dependent transcriptome reveals high-level regulatory architecture in *Bacillus subtilis*. *Science* 335 1103–1106. 10.1126/science.1206848 22383849

[B42] Otwinowski Z. Minor W. (1997). Processing of X-ray diffraction data collected in oscillation mode. *Macromol. Crystallogr.* 276 307–326. 10.1016/S0076-6879(97)76066-X 27754618

[B43] Pallares I. Iglesias V. Ventura S. (2015). The rho termination factor of clostridium botulinum contains a prion-like domain with a highly amyloidogenic core. *Front. Microbiol.* 6:1516. 10.3389/fmicb.2015.01516 26779170 PMC4703818

[B44] Pedersen J. S. Andersen C. B. Otzen D. E. (2010). Amyloid structure–one but not the same: the many levels of fibrillar polymorphism. *FEBS J.* 277 4591–4601. 10.1111/j.1742-4658.2010.07888.x 20977663

[B45] Pouplana S. Espargaro A. Galdeano C. Viayna E. Sola I. Ventura S. (2014). Thioflavin-S staining of bacterial inclusion bodies for the fast, simple, and inexpensive screening of amyloid aggregation inhibitors. *Curr. Med. Chem.* 21 1152–1159. 24059241 10.2174/09298673113206660256

[B46] Quay D. H. Cole A. R. Cryar A. Thalassinos K. Williams M. A. Bhakta S. (2015). Structure of the stationary phase survival protein YuiC from *B.* s*ubtilis*. *BMC Struct. Biol.* 15:12. 10.1186/s12900-015-0039-z 26163297 PMC4499186

[B47] Ramachandran G. N. Ramakrishnan C. Sasisekharan V. (1963). Stereochemistry of polypeptide chain configurations. *J. Mol. Biol.* 7 95–99. 13990617 10.1016/s0022-2836(63)80023-6

[B48] Rambo R. P. Tainer J. A. (2013). Accurate assessment of mass, models and resolution by small-angle scattering. *Nature* 496 477–481. 10.1038/nature12070 23619693 PMC3714217

[B49] Rinas U. Garcia-Fruitos E. Corchero J. L. Vazquez E. Seras-Franzoso J. Villaverde A. (2017). Bacterial inclusion bodies: discovering their better half. *Trends Biochem. Sci.* 42 726–737. 10.1016/j.tibs.2017.01.005 28254353

[B50] Romero D. Aguilar C. Losick R. Kolter R. (2010a). Amyloid fibers provide structural integrity to Bacillus subtilis biofilms. *Proc. Natl. Acad. Sci. U.S.A.* 107 2230–2234. 10.1073/pnas.0910560107 20080671 PMC2836674

[B51] Romero D. Aguilar C. Losick R. Kolter R. (2010b). Amyloid fibers provide structural integrity to *Bacillus subtilis* biofilms. *Proc. Natl. Acad. Sci. U.S.A.* 107 2230–2234. 10.1073/pnas.0910560107 20080671 PMC2836674

[B52] Saeed S. M. Fine G. (1967). Thioflavin-T for amyloid detection. *Am. J. Clin. Pathol.* 47 588–593. 4164576 10.1093/ajcp/47.5.588

[B53] Sanders K. Lin C. L. Smith A. J. Cronin N. Fisher G. Eftychidis V. (2017). The structure and function of an RNA polymerase interaction domain in the PcrA/UvrD helicase. *Nucleic Acids Res.* 45 3875–3887. 10.1093/nar/gkx074 28160601 PMC5397179

[B54] Schneidman-Duhovny D. Hammel M. Sali A. (2010). FoXS: a web server for rapid computation and fitting of SAXS profiles. *Nucleic Acids Res.* 38 W540–W544. 10.1093/nar/gkq461 20507903 PMC2896111

[B55] Schuck P. (2000). Size-distribution analysis of macromolecules by sedimentation velocity ultracentrifugation and lamm equation modeling. *Biophys. J.* 78 1606–1619. 10.1016/S0006-3495(00)76713-0 10692345 PMC1300758

[B56] Sormanni P. Aprile F. A. Vendruscolo M. (2015a). The CamSol method of rational design of protein mutants with enhanced solubility. *J. Mol. Biol.* 427 478–490. 10.1016/j.jmb.2014.09.026 25451785

[B57] Sormanni P. Camilloni C. Fariselli P. Vendruscolo M. (2015b). The s2D method: simultaneous sequence-based prediction of the statistical populations of ordered and disordered regions in proteins. *J. Mol. Biol.* 427 982–996. 10.1016/j.jmb.2014.12.007 25534081

[B58] Sulatskaya A. I. Rodina N. P. Povarova O. I. Kuznetsova I. M. Turoverov K. K. (2017). Different conditions of fibrillogenesis cause polymorphism of lysozyme amyloid fibrils. *J. Mol. Struct.* 1140 52–58. 10.1016/j.molstruc.2016.10.037

[B59] Tompa P. (2009). Structural disorder in amyloid fibrils: its implication in dynamic interactions of proteins. *FEBS J.* 276 5406–5415. 10.1111/j.1742-4658.2009.07250.x 19712107

[B60] Tuteja N. Tuteja R. (2004). Prokaryotic and eukaryotic DNA helicases. Essential molecular motor proteins for cellular machinery. *Eur. J. Biochem.* 271 1835–1848. 10.1111/j.1432-1033.2004.04093.x 15128294 PMC7164108

[B61] Volkov V. V. Svergun D. I. (2003). Uniqueness of ab-initio shape determination in small-angle scattering. *J. Appl. Crystallogr.* 36 860–864. 10.1107/S1399004715002576 27630371 PMC5023043

[B62] Walsh I. Seno F. Tosatto S. C. Trovato A. (2014). PASTA 2.0*:* an improved server for protein aggregation prediction. *Nucleic Acids Res.* 42 W301–W307. 10.1093/nar/gku399 24848016 PMC4086119

[B63] Werner F. (2008). Structural evolution of multisubunit RNA polymerases. *Trends Microbiol* 16 247–250. 10.1016/j.tim.2008.03.008 18468900

[B64] Wiedermannova J. Sudzinova P. Koval T. Rabatinova A. Sanderova H. Ramaniuk O. (2014). Characterization of HelD, an interacting partner of RNA polymerase from *Bacillus subtilis*. *Nucleic Acids Res.* 42 5151–5163. 10.1093/nar/gku113 24520113 PMC4005671

[B65] Wolfe K. J. Cyr D. M. (2011). Amyloid in neurodegenerative diseases: friend or foe? *Semin. Cell Dev. Biol.* 22 476–481. 10.1016/j.semcdb.2011.03.011 21458579 PMC3182296

[B66] Yang W. (2010). Lessons learned from UvrD helicase: mechanism for directional movement. *Annu. Rev. Biophys.* 39 367–385. 10.1146/annurev.biophys.093008.131415 20192763 PMC3480338

[B67] Yuan A. H. Hochschild A. (2017). A bacterial global regulator forms a prion. *Science* 355 198–201. 10.1126/science.aai7776 28082594 PMC5460984

