# Supplementary material for: Bacillus subtilis HelD, an RNA Polymerase Interacting Helicase, Forms Amyloid-Like Fibrils
Source: Front Microbiol. 2018 Aug 21;9:1934. doi: 10.3389/fmicb.2018.01934 (PMC6111841; doi:10.3389/fmicb.2018.01934)
Supplement: Supplementary file 1 [file Data_Sheet_1.docx]

**Supplementary Data**

***Bacillus subtilis* HelD, an RNA polymerase interacting helicase, forms amyloid-like fibrils**

Gundeep Kaur^1^, Srajan Kapoor^1^ and Krishan Gopal Thakur^1#^

^1^G. N. Ramachandran Protein Centre, Structural Biology Laboratory, Council of Scientific and Industrial Research-Institute of Microbial Technology (CSIR-IMTECH), Chandigarh-160036, India

^#^**Correspondence**

Krishan Gopal Thakur

Council of Scientific and Industrial Research-Institute of Microbial Technology,

Chandigarh-160036, India.

E-mail: [krishang@imtech.res.in](mailto:krishang@imtech.res.in)

**Running Title:** HelD forms amyloids *in vitro* and *in vivo*

**Keywords:** *Bacillus subtilis*, Amyloid fibrils, intracellular amyloids, HelD, SEC-SAXS, oligomerization


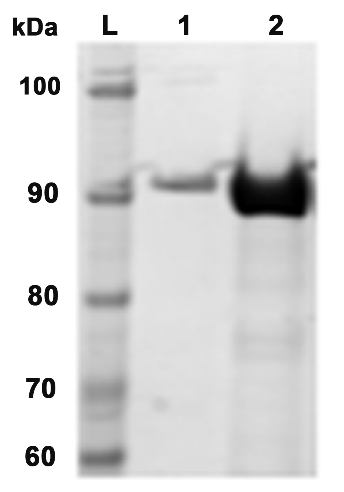


**Supplementary Figure 1:** 15% SDS-PAGE profile showing elution fractions (Lane 1 & 2) of HelD after Co-NTA affinity chromatography.





**Supplementary Figure 2:** Thermal melting profile of HelD upon heating (black) and cooling (blue) measured using far-UV CD at 222 nm. The data fitting (magenta curve) was performed using OriginPro 2016.





**Supplementary Figure 3:** The SEC profile of native and heated sample of HelD. The protein sample was diluted in the 10 mM sodium phosphate buffer and heated up to 95 °C followed by cooling back to 20 °C. SEC data suggests that upon heating HelD forms either higher order oligomers or soluble aggregates that elute in the void volume.


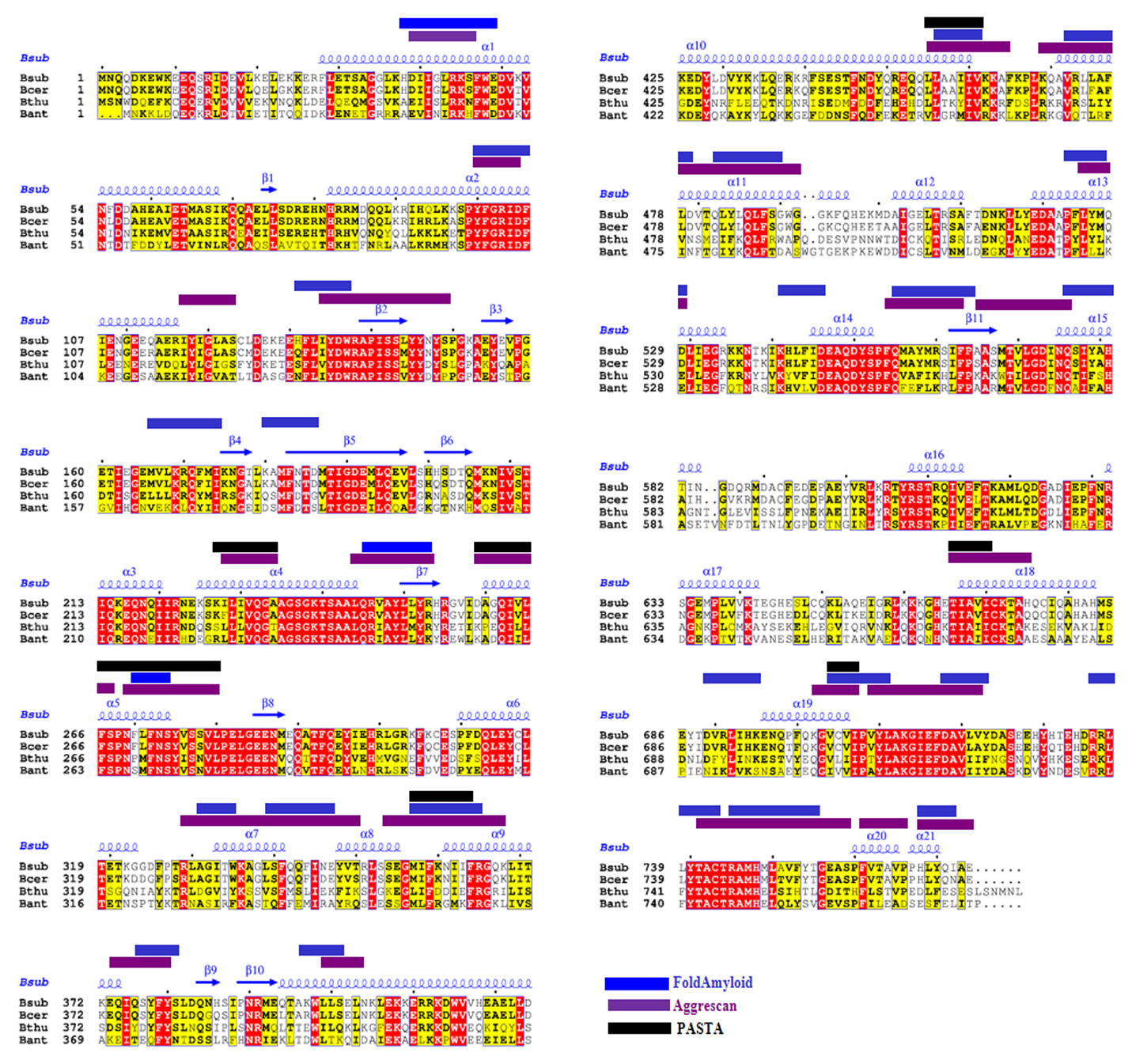


**Supplementary Figure 4:** Multiple sequence alignment of HelD homologs performed using ClustalW ([Larkin et al., 2007](#_ENREF_1)). The secondary structure of *B. subtilis*  HelD was predicted using PSIPRED ([McGuffin et al., 2000](#_ENREF_2)) and the final alignment figure along with representation of secondary structures was prepared using ESPript ([Robert and Gouet, 2014](#_ENREF_3)). Blue (FoldAmyloid), purple (Aggrescan) and black (PASTA) rectangular boxes represent the predicted amyloidogenic regions spanning all along the sequence in *B. subtilis*  HelD. The predicted amyloidogenic regions are lying mostly in the highly conserved regions of Bacillus species. Abbreviations used are Bsub, *B. subtilis*, Bant, *B. anthracis*, Bthu, B. *thuringiensis* and Bcer, *B. cereus.*

**
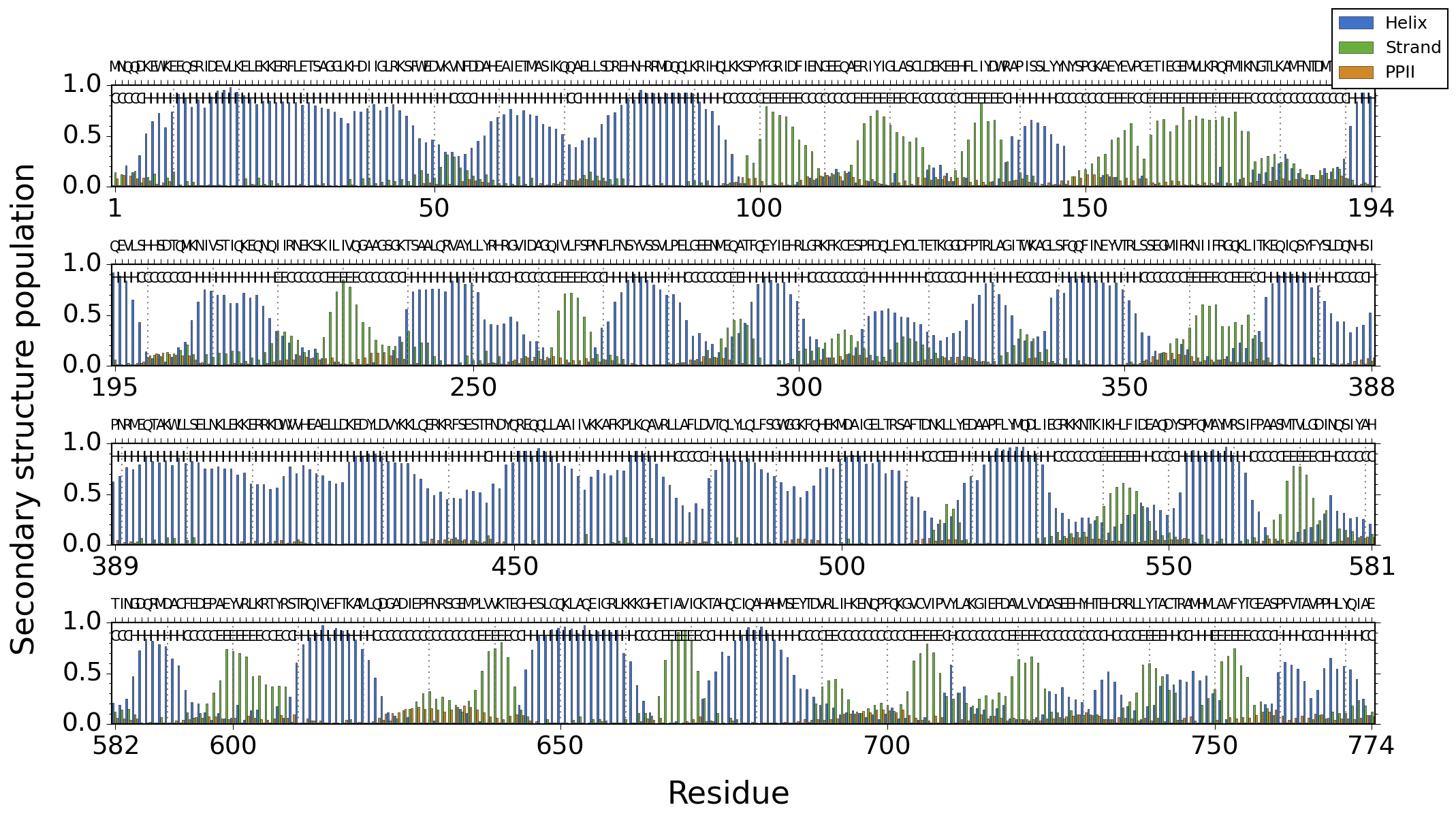
**

**Supplementary Figure 5:** s2D ([Sormanni et al., 2015](#_ENREF_4)) based prediction of polyproline II regions in the *B. subtilis* HelD.

**
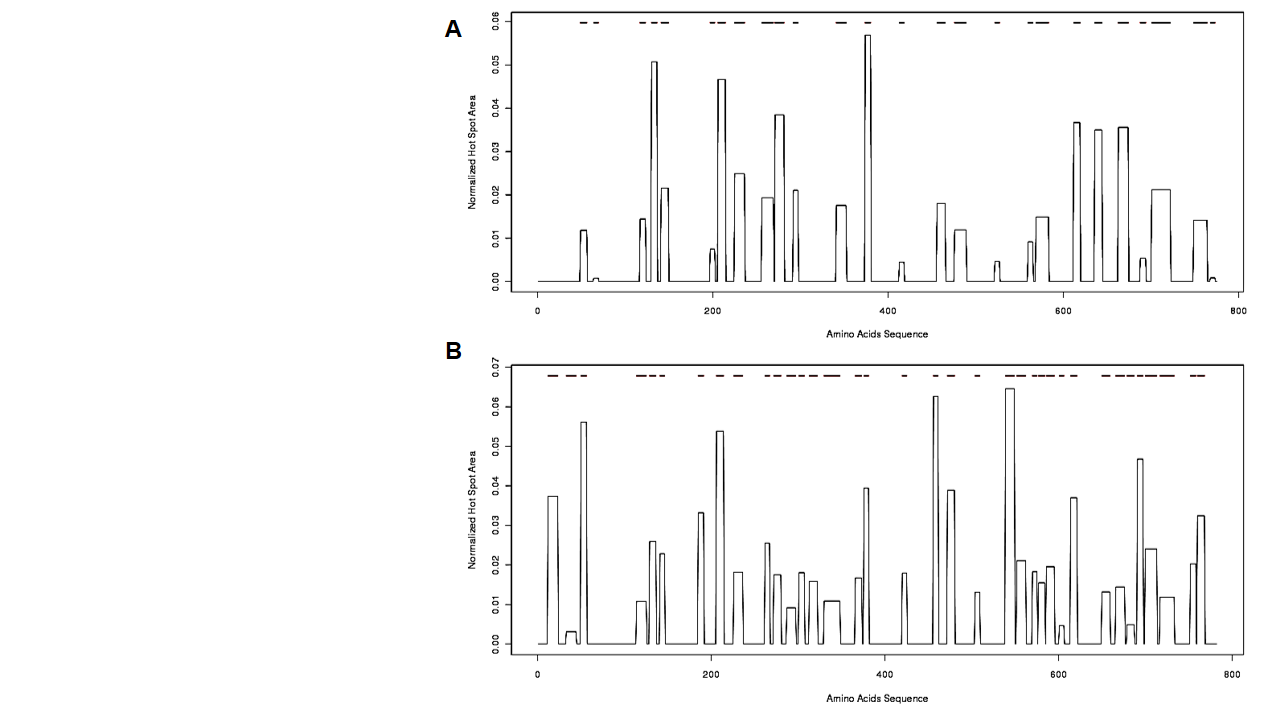
** **
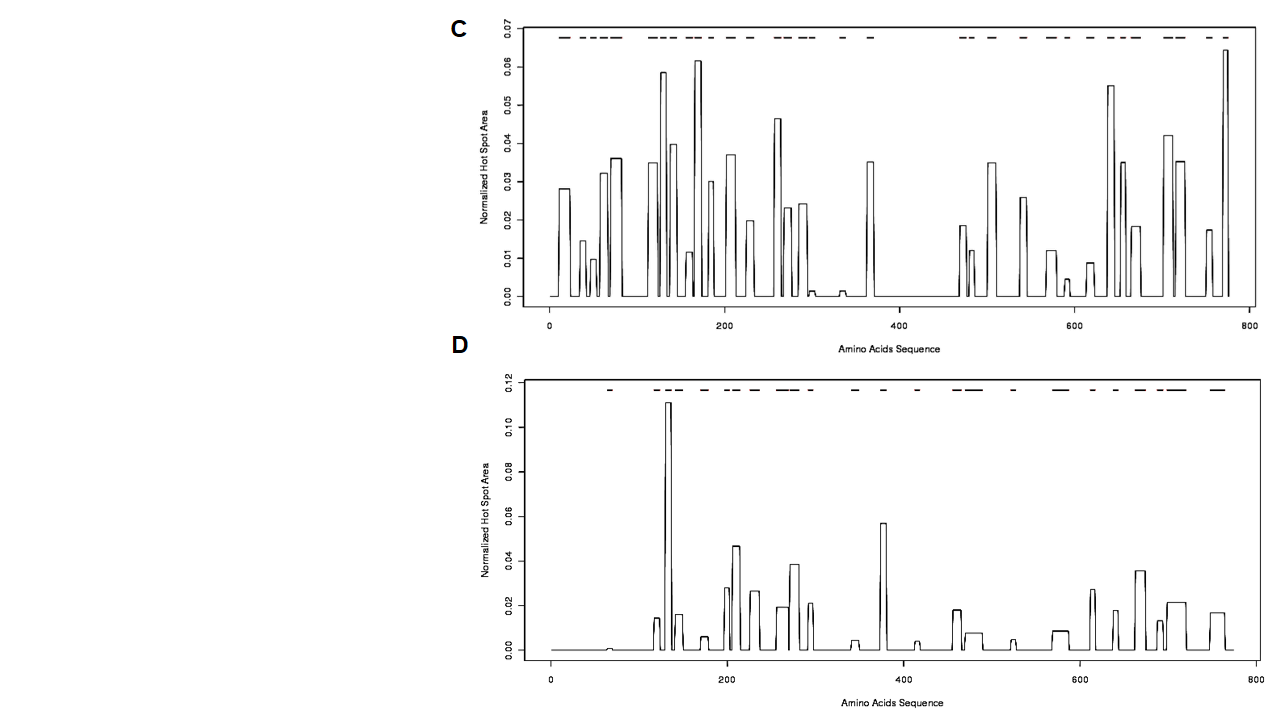
**

*B. subtilis* HelD

Number of hot spots: 26

*B. thuringiensis* HelD

Number of hot spots: 36

*B. anthracis* HelD

Number of hot spots: 33

*B. cereus* HelD

Number of hot spots: 24

**Supplementary Figure 6:** MetAmyl based prediction of amyloidogenic regions in the HelD homologs. The number of hot spots predicted in the HelD from pathogenic species is higher compared to *B. subtilis*.

**References:**

Larkin, M.A., Blackshields, G., Brown, N.P., Chenna, R., McGettigan, P.A., McWilliam, H., Valentin, F., Wallace, I.M., Wilm, A., Lopez, R., Thompson, J.D., Gibson, T.J., and Higgins, D.G. (2007). Clustal W and Clustal X version 2.0. *Bioinformatics* 23**,** 2947-2948. doi: 10.1093/bioinformatics/btm404.

McGuffin, L.J., Bryson, K., and Jones, D.T. (2000). The PSIPRED protein structure prediction server. *Bioinformatics* 16**,** 404-405.

Robert, X., and Gouet, P. (2014). Deciphering key features in protein structures with the new ENDscript server. *Nucleic Acids Res* 42**,** W320-324. doi: 10.1093/nar/gku316.

Sormanni, P., Camilloni, C., Fariselli, P., and Vendruscolo, M. (2015). The s2D method: simultaneous sequence-based prediction of the statistical populations of ordered and disordered regions in proteins. *J Mol Biol* 427**,** 982-996. doi: 10.1016/j.jmb.2014.12.007.
